# The Effect of Anxiety and Depression on Sleep Quality of Individuals With High Risk for Insomnia: A Population-Based Study

**DOI:** 10.3389/fneur.2019.00849

**Published:** 2019-08-13

**Authors:** Chang-Myung Oh, Ha Yan Kim, Han Kyu Na, Kyoo Ho Cho, Min Kyung Chu

**Affiliations:** ^1^Division of Endocrinology and Metabolism, CHA Bundang Medical Center, School of Medicine CHA University, Seongnam-si, South Korea; ^2^Biostatistics Collaboration Unit, Yonsei University College of Medicine, Seoul, South Korea; ^3^Department of Neurology, Yonsei University College of Medicine, Seoul, South Korea

**Keywords:** insomnia, mood disorder, depression, anxiety, sleep quality

## Abstract

**Introduction:** One of the most common sleep disorders, insomnia is a significant public health concern. Several psychiatric disorders, such as anxiety disorders and depression, have shown strong relationships with insomnia. However, the clinical impact of the combination of these two conditions on insomnia severity and sleep quality remains unknown. We investigated the relationship between sleep disturbance and psychiatric comorbidities in subjects with high risk for insomnia.

**Methods:** We analyzed data from a nation-wide cross-sectional survey of Korean adults aged 19 ~ 69 years conducted from November 2011 to January 2012. The survey was performed via face-to-face interviews using a structured questionnaire. We used the insomnia severity index (ISI) to evaluate insomnia and defined respondents with ISI scores of ≥10 were considered to be at high risk for insomnia. To diagnose anxiety and depression, we used the Goldberg anxiety scale (GAS) and Patient Health Questionnaire-9 (PHQ-9), respectively.

**Results:** Of the 2,762 respondents, 290 (10.5%) were classified as subjects with high risk for insomnia; anxiety [odds ratio (OR), 9.8; 95% confidence interval (CI), 7.3–13.1] and depression (OR, 19.7; 95% CI, 13.1–29.6) were more common in this population than in participants without insomnia. Of the participants with insomnia, 152 (52.4%) had neither anxiety nor depression, 63 (21.7%) only had anxiety, 21 (7.2%) only had depression, and 54 (18.6%) had both anxiety and depression. The group with both anxiety and depression was associated with worse scores on sleep-related scales than the other groups [high ISI, Pittsburgh Sleep Quality Index (PSQI), and Epworth Sleepiness Scale]. The relationship between outcome measures (ISI and PSQI) and psychiatric problems was significant only when anxiety and depression were present. The PSQI has a significant mediation effect on the relationship between psychiatric comorbidities and insomnia severity.

**Conclusion:** Among the respondents with insomnia, psychiatric comorbidities may have a negative impact on daytime alertness, general sleep quality, and insomnia severity, especially when the two conditions are present at the same time. Clinicians should, therefore, consider psychiatric comorbidities when treating insomnia.

## Introduction

As one of the most common sleep disorders, insomnia has become a significant public health problem. While the prevalence of insomnia varies considerably across countries, its global prevalence of insomnia as defined by the Diagnostic and Statistical Manual of Mental Disorders, Fourth Edition (DSM-IV) criteria is estimated to be ~6–10% (1–3).

Insomnia reportedly increases the rate of car accidents (4), decreases job performance, results in self-medication with alcohol as well as socio-economic problems (5–7), and has been associated with the onset of cardiovascular diseases (8).

Although a previous (second) edition of the International Classification of Sleep Disorders (ICSD-2) distinguished psychophysiological insomnia from mental-illness induced insomnia (9), there are substantial overlap features between the two: e.g., conditioned arousal, poor sleep hygiene, and excessive worry about sleep (10). Also, many subjects with insomnia have psychiatric comorbidities, rendering the discrimination of insomnia subtypes difficult. Because of these issues (overlapping primary and secondary insomnia), in the next version, ICSD-3, insomnia was recategorized according to time course (10). Nevertheless, it is still of great value to recognize the most common psychiatric comorbidities such as anxiety disorders and depression in patients with insomnia (11). Among patients with insomnia, the prevalence of anxiety disorder, including generalized anxiety disorder, panic disorder, post-traumatic stress disorder, and phobia, is 24–36% (11, 12), while that of major depression is 14–31% (11, 12). Conversely, about 90% of patients with depression complain of sleep disturbance (13, 14). Similarly, sleep problems are much more common among individuals with anxiety disorders (15, 16).

Multiple studies have investigated the relationship between sleep disorders and psychiatric comorbidity. A study that used data collected by the national survey in the United States revealed that individuals with any comorbid sleep problem (especially non-restorative sleep) are more prone to impairments of daytime activities (17). Using data obtained by the same survey, another investigation reported that the rate of insomnia complaints was highest among individuals with anxiety and mood disorders (42–63%) (18). However, to date, it remains unclear whether the combination of anxiety and mood (depression) problems aggravate the severity of insomnia itself and the sleep quality of people at high risk for insomnia.

This study explored the relationships among insomnia severity, sleep quality and daytime sleepiness, and comorbidities with anxiety and depression among individuals at high risk for insomnia. In addition, we used mediation modeling to evaluate whether insomnia severity is mediated by changes in general sleep quality.

## Methods

### Survey Procedure

We used data from a nationwide, cross-sectional survey of headache and anxiety in the general Korean population conducted from November 2011 to January 2012 (19). Trained interviewers performed structured, face-to-face interviews that included questionnaires regarding sleep and headache disorders and mood problems (anxiety and depression). Adults between the ages of 19 and 69 years were included. Besides, we collected the respondents' demographic and geographic information. The target area, sampling method, and detailed survey procedures were same as the previously documented process (19). The distribution in this study and the total population were not significantly different in sex, age groups, size of a residential area, and educational level.

### Diagnosis of Insomnia, Anxiety, and Depression

We used the insomnia severity index (ISI) to define high risk for insomnia. Subjects who received an ISI score of ≥10 were classified as high risk for insomnia according to a previous community-based study (20). Using an ISI score of 10 as the cutoff, insomnia was detected with 86.1% sensitivity and 87.7% specificity (20).

For the diagnosis of anxiety and depression, we used the Goldberg anxiety scale (GAS) and Patient Health Questionnaire-9 (PHQ-9), respectively. The GAS consists of four screening and five supplementary questions. The validated Korean version of the GAS features a sensitivity of 82.0% and a specificity of 94.4% (21). At least two positive answers to screening and five or more positive answers to complementary questions in GAS questions indicated anxiety. The PHQ-9 was used to diagnose depression (22). The Korean version of the PHQ-9 features 81% sensitivity and 89.9% specificity (23). Participants with a PHQ-9 score of ≥10 were considered to have depression. We classified the respondents into four groups: individuals (1) without anxiety or depression, (2) with anxiety but without depression, (3) without anxiety but with depression, and (4) with both anxiety and depression. The demographic data of the participants are shown in Table 1.

**Table 1:** Sociodemographic distribution of all survey participants, the total Korean population, and of cases identified as insomnia, anxiety, and depression.

	**Insomnia (*n* = 290)**	***P***	**Anxiety (*n* = 268)**	***P***	**Depression, (*n* = 116)**	***P***
Gender		<0.01		<0.01		<0.01
Male (1,345)	117 (8.7%)		109 (8.1%)		43 (3.2%)	
Female (1,350)	173 (12.8%)		159 (11.8%)		73 (5.4%)	
Age		0.53		0.71		0.75
19–29 (542)	59 (10.9%)		53 (9.8%)		23 (4.2%)	
30–39 (604)	53 (8.8%)		51 (8.4%)		32 (5.3%)	
40–49 (611)	66 (10.8%)		67 (11.0%)		24 (3.9%)	
50–59 (529)	63 (11.9%)		53 (10.0%)		22 (4.2%)	
60–69 (409)	49 (12.0%)		44 (10.8%)		15 (3.7%)	
Size of residential area		0.95		0.71		0.76
Large city (1,248)	136 (10.9%)		130 (10.4%)		57 (4.6%)	
Medium-to-small city (1,186)	125 (10.5%)		112 (9.4%)		47 (4.0%)	
Rural are (261)	29 (11.1%)		26 (10.0%)		12 (4.6%)	
Educational level^*^		<0.01		0.03		0.70
Middle school or less (393)	62 (15.8%)		55 (14.0%)		20 (5.1%)	
High school (1,208)	116 (9.6%)		111 (9.2%)		49 (4.1%)	
College or more (1,068)	109 (10.2%)		100 (9.4%)		47 (4.4%)	
Type of job^**^		0.58		0.57		0.32
Shift work (145)	18 (12.4%)		17 (11.7%)		9 (6.2%)	
Regular work (2,157)	224 (10.4%)		209 (9.7%)		87 (4.0%)	

Total subjects: 2,695; P, p-value;

* non-responder: 26;

** *non-responder: 393*.

### Measures Related to Sleep Quality

Each individual was asked to complete questionnaires, including the Pittsburgh Sleep Quality Index (PSQI), which accesses the multifactorial construct of sleep dysfunction with strong reliability and validity (24), and the Epworth Sleepiness Scale (ESS). which assesses each participant's daytime sleepiness (25). The subjective and objective sleep qualities measured with ISI feature a high concordance with daytime disability (26). Even young adults with insomnia frequently complain of daytime sleepiness (27). Additionally, each subject's ISI score was categorized into one of three groups (mild insomnia, 10 ≤ ISI < 15; moderate, 15 ≤ ISI < 20; severe, ISI > 20).

### Ethics

This study was approved by the institutional review board/ethics committee of the Severance Hospital, and written informed consent was obtained from each participant.

### Statistical Analysis

We used the chi-square test to examine whether the number of diagnoses differed according to sex, age, size of a residential area, educational level, and types of work shift. We compared group differences in sleep time and ESS and PSQI scores using the Kruskal-Wallis test followed by Bonferroni's multiple comparison correction. Group differences in ISI severity and insomnia characteristics were compared with Jonckheere's trend test. Univariate and multivariate logistic regression analysis was performed to evaluate the odds ratio (OR) of anxiety and depression in individuals with high risk for insomnia compared to those without high risk for insomnia. The same procedure was used to compare the PSQI and ISI scores stratified by the presence of depression or anxiety between groups; sex, age group, size of residential area, and education level were included in the multivariate analysis. To examine whether the association between anxiety and depression comorbidities and insomnia risk is mediated by sleep quality, we performed a mediation analysis based on a previously developed method (28). In all the statistical analyses, two-tailed *p* <0.05 were considered statistically significant.

## Results

### Demographic Data of the Subjects

**Figure 1:**
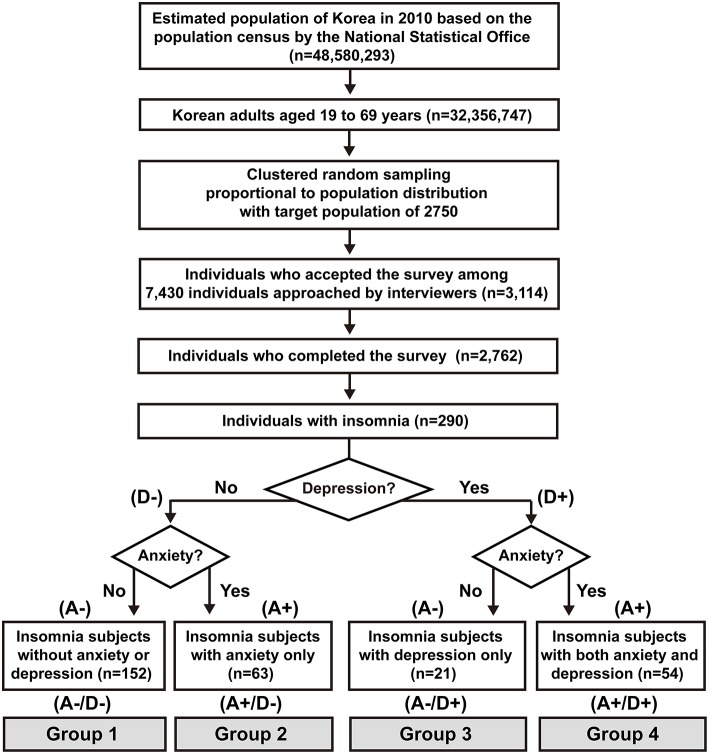
Flow chart of participants in the Korean Headache-Sleep study. Whether individuals had anxiety (A) or depression (D) are denoted by positive (+) and negative (–). A–/D–, without anxiety or depression; A+/D–, with anxiety only; A–/D+, with depression only; A+/D+, with both anxiety and depression.

Of the total 7,430 interviewees, 3,114 completed the survey (acceptance rate of 41.9%), and 352 subjects suspended the interview. The final sample that completed the survey and the interview included 2,762 individuals (19). The enrollment flow chart of this study is presented in Figure 1. The prevalence of insomnia, anxiety, and depression according to sex, age distribution, size of the residential area, and education level is depicted in Table 1. Insomnia, depression, and anxiety were significantly more prevalent among women. Participants with the lowest educational levels were associated with a higher prevalence of insomnia and anxiety than were those who had completed high school, college, or graduate school.

### Prevalence of Insomnia, Anxiety, and Depression and Mean Scores on Sleep-Related Scales

Of the 2,762 participants, 290 (10.5%) were classified as having a high risk for insomnia. The frequencies of insomnia, anxiety, and depression are presented in Table 1 according to sex, age group, size of the residential area, and education level. The mean ISI, ESS, and PSQI scores of individuals with high risk for insomnia were 14.37 ± 4.39, 7.73 ± 4.87, and 7.42 ± 2.59, respectively. Of the 290 subjects with high risk for insomnia, 152 (52.4%) had neither anxiety nor depression (Group 1), 63 (21.7 %) had anxiety only (Group 2), 21 (7.2%) had depression only (Group 3), and 54 (18.6%) had both anxiety and depression (Group 4). Anxiety was more common among individuals who had a high risk for insomnia than among those who did not [40.3% vs. 6.1%; OR, 10.1; 95% confidence interval (CI), 7.6–13.4]; this finding remained consistent after adjustment for demographic variables (OR, 9.8; 95% CI, 7.3–13.1). Depression was also more common among individuals who had a high risk for insomnia than those who did not (25.9% vs. 1.7%; OR, 20.1; 95% CI, 13.4–30.2); this finding also remained consistent after adjustment for demographic variables (OR, 19.7l 95% CI, 13.1–29.6) (Table 2).

**Table 2:** Logistic regression analysis.

	**Insomnia, *n* = 290**	**Non-insomnia, *n* = 2,472**	**Univariate analysis**		**Multivariate analysis^¶^**	
			**OR**	**CI**	**OR**	**CI**
Anxiety	117 (40.3%)	151 (6.1%)	10.1	7.6–13.4	9.8	7.3–13.1
Depression	75 (25.9%)	41 (1.7%)	20.1	13.4–30.2	19.71	13.1–29.6

Insomnia vs. non-insomnia population. OR, odds ratio; CI, 95% confidence interval;

¶ *adjusted for age, gender, education level, and size of residential area*.

### Difference in Sleep-Related Scales Among the Four Groups

**Figure 2:**
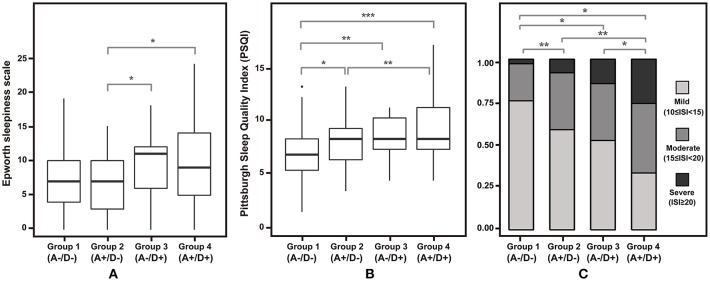
Differences in scores on sleep-related scale across four groups. **(A)** Epworth sleepiness scale and **(B)** Pittsburg sleep quality index is depicted in box plots. **(C)** Proportion of each insomnia severity index category is shown in a stacked bar plot. A–/D–, without anxiety or depression; A+/D–, with anxiety only; A–/D+, with depression only; A+/D+, with both anxiety and depression; ^*^p < 0.05, ^**^p < 0.01, and ^***^p < 0.001.

**Figure 3:**
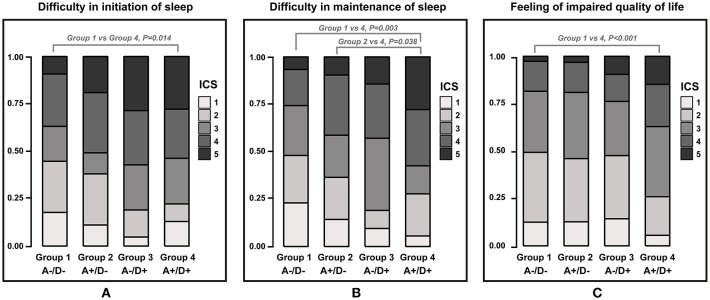
Stacked bar plot showing the proportion of each insomnia characteristic scale 1–5 across groups. A–/D–, without anxiety or depression; A+/D–, with anxiety only; A–/D+, with depression only; A+/D+, with both anxiety and depression. Insomnia characteristic scale (ICS) was graded on a scale of 1–5.

Participants at high risk for insomnia with both anxiety and depression were associated with significantly higher ESS and PSQI scores relative to the anxiety-only group (Figure 2). Meanwhile, individuals with no psychiatric comorbidities were associated with lower PSQI scores relative to the three other groups. ISI scores were significantly higher among participants with both anxiety and depression than those with any psychiatric comorbidities. When stratified by the presence of anxiety and depression, severe ISI tended to increase from Groups 1–4 (Figure 3).

### Insomnia Characteristics According to the Presence of Anxiety or Depression

No statistical differences were observed among the 4 groups in average sleep time during workdays, weekends, and overall sleep. The three insomnia-related symptom scales of each group are presented in Figure 3. Regarding the difficulty in initiating sleep and feelings of impaired quality of life, only Groups 1 and 4 featured a statistically significant difference. Group 4 was more likely to have difficulties in maintaining sleep than were Groups 1 and 2. The groups did not differ in their scores on the frequent awakening scale.

### Mediation Model Analysis

**Figure 4:**
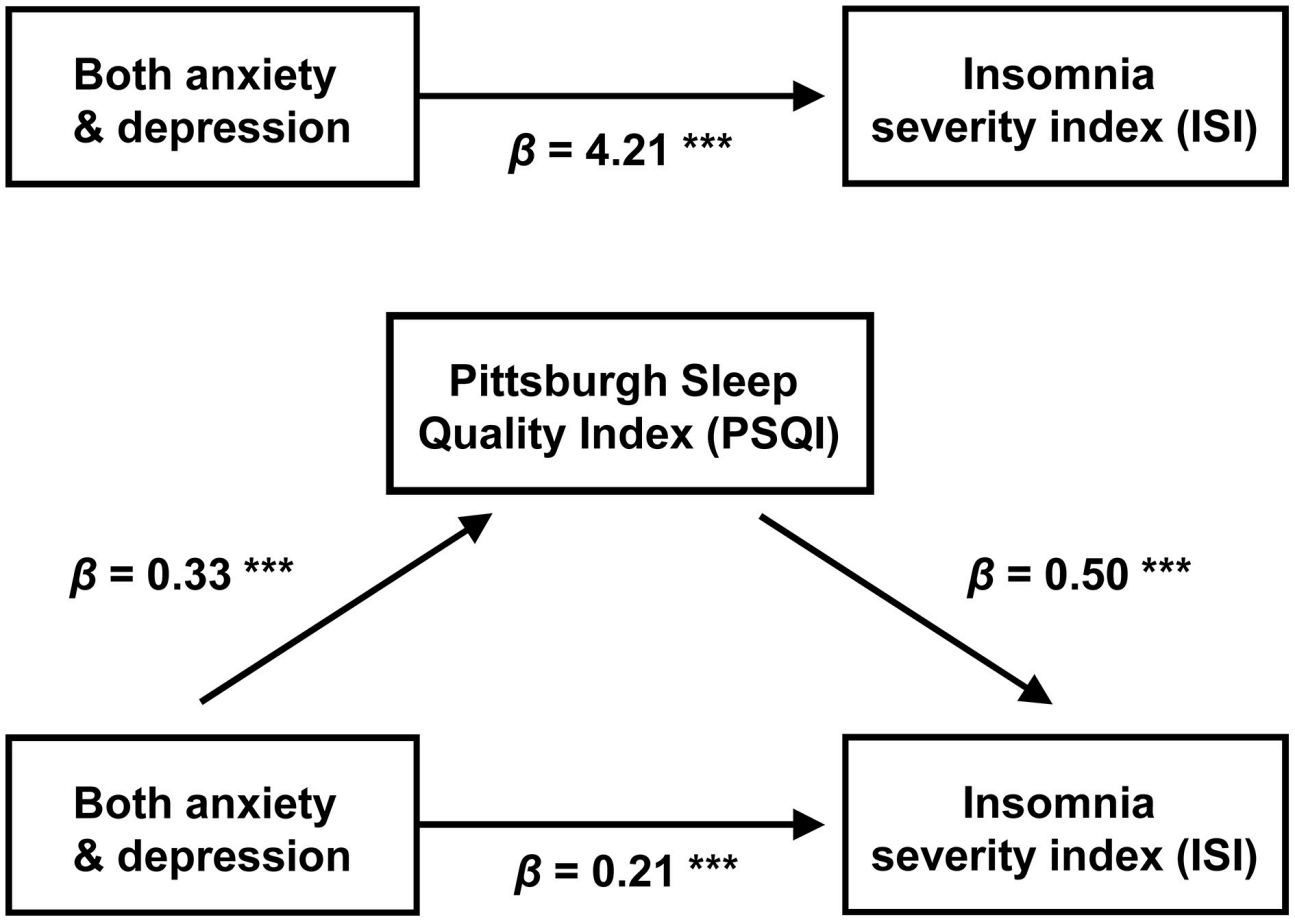
Mediation model: the indirect effect of combined anxiety and depression on ISI scores through PSQI. Unstandardized regression coefficients are presented for each path ^***^p < 0.001.

When insomnia was comorbid with both anxiety and depression, PSQI and ISI were significantly correlated with each other (Tables 3, 4). The relationship between anxiety and ISI (beta = 4.21) was attenuated when PSQI was used as a mediator (Figure 4). The Sobel test revealed the significance of the indirect effect of PSQI in mediating both anxiety and depression and ISI (Table 5).

**Table 3:** Mediation analysis.

**Variables**		**Beta**	***SE***	***P***
**Outcome**	**Predictors**			
ISI	PSQI	0.50	0.08	<0.0001
ISI	Both anxiety and depression	0.21	0.56	<0.0001
PSQI	Both anxiety and depression	0.33	0.37	<0.0001
Indirect effect	0.17	0.36	<0.0001	
Total effect	0.37	0.61	<0.0001	

*ISI, insomnia severity index; PSQI, Pittsburgh Sleep Quality Index; SE, standard error; P, p-value*.

**Table 4:** Linear regression analysis, endpoint: ISI.

**Univariate analysis**	**Beta**	***SE***	***P***	**Multivariate analysis^**¶**^**	**Beta**	***SE***	***P***
Anxiety only	0.2	0.6	0.70	Anxiety only	0.2	0.6	0.75
Depression only	0.8	1.0	0.40	Depression only	1.0	1.0	0.30
only one of anxiety or depression	0.5	0.6	0.41	Only one of anxiety or depression	0.5	0.6	0.38
Anxiety + Depression	4.2	0.6	<0.0001	Anxiety + Depression	4.1	0.6	<0.0001

ISI, insomnia severity index;

¶ *adjusted for age, gender, education level, and size of residential area; SE, standard error; P, p-value*.

**Table 5:** Linear regression analysis, endpoint: PSQI.

**Univariate analysis**	**Beta**	***SE***	***P***	**Multivariate analysis^**¶**^**	**Beta**	***SE***	***P***
Anxiety only	0.1	0.4	0.85	Anxiety only	0.1	0.4	0.86
Depression only	0.9	0.6	0.11	Depression only	1.0	0.6	0.10
Only one of anxiety or depression	0.4	0.3	0.28	Only one of anxiety or depression	0.4	0.3	0.27
Anxiety + Depression	4.1	0.3	<0.0001	Anxiety + Depression	2.2	0.4	<0.0001

PSQI, Pittsburgh Sleep Quality Index;

¶ *adjusted for age, gender, education level, and size of residential area; SE, standard error, P, p-value*.

## Discussion

We found the prevalence of high risk for insomnia in the Korean adult population to be 10.5%, which is significantly different from previously reported rates in the Korean population (about 20%). Differences between the methodology of our study and those of previous investigations, which used telephone-based interviews without validated questionnaires, may account for the divergent findings (29, 30). Measuring residential noise levels in addition to employing a structured questionnaire, a population-based study conducted in Japan, reported a crude prevalence of insomnia of 8.8% (31), which better accords with our results. The prevalence of overall anxiety and depression observed in our study was also similar to or slightly higher than the rates reported by prior Korean epidemiologic studies (32, 33). Similar to a previous study (34), we found that the prevalence of insomnia to be higher among women and individuals with low levels of education. We also found no association between age and the prevalence of insomnia. This discrepancy may be due to the changes in the lifestyles of young Korean adults, which might cause higher rates of insomnia among adults below the age of 30 (35).

We observed that 47.6% of individuals with high risks for insomnia had comorbidities of anxiety or depression. This finding in the Korean population was similar to a previously reported rate: 40% of individuals with insomnia had comorbid psychiatric disorders (11), among which depression and anxiety disorders were reportedly the most common.

As expected, Group 1 had lower PSQI and ISI scores than those with anxiety-only, depression-only, or both (Groups 2, 3, and 4). The ESS scores of subjects with anxiety-only or depression-only (Groups 2 and 3), but not both (Group 4), did not differ significantly from those of individuals without any anxiety or depression (Group 1) as depicted in Figure 1. This might be due to individuals with insomnia having excessive hyperarousal continuing throughout the day time rather than daytime sleepiness. Group 4 was consistently associated with poorer sleep-related indices than was Group 2. While Groups 3 and 4 differed significantly in the proportion of participants who had higher ISI scores (Figure 3C), they did not differ significantly in ESS and PSQI scores. This result might be due to a lack of statistical power associated with sample sizes (Group 3 was relatively small).

Individuals with high risk for insomnia are associated with an increased incidence of anxiety and depression relative to those without insomnia. After controlling for possible confounding variables, subjects with insomnia were found to be 9.8 times more likely to have anxiety than subjects without insomnia and 19.7 times more likely to have depression. In addition, we found Groups 2, 3, and 4, in order, had significantly higher rates of insomnia symptoms.

Numerous studies have reported an association between insomnia and depression and anxiety (36–40). Indeed, patients with persistent insomnia are reportedly predisposed to developing psychiatric illness and are more prone to the recurrence of depression (41, 42). Among patients with partially treated depression, residual symptoms such as anxiety and insomnia are among the most powerful predictors for relapse (41). Additionally, insomnia and anxiety share a pathogenetic mechanism: hyperarousal caused by dysregulation of neurotransmitter systems including cholinergic and GABA (gamma-aminobutyric acid) eric mechanisms (43). Hyperarousal and insufficient sleep disrupt the function of corticolimbic circuitry, which leads to impaired affective reactivity and regulation (44). Genetic studies also showed a strong overlap between genetic influences on insomnia, depression, and anxiety (44). This is reflected in high comorbidity of the two conditions (70–90% of patients with anxiety report insomnia) (45).

The mediational model revealed that the effect of combined anxiety and depression on insomnia severity is mediated by poor sleep behaviors. Considering that the essential feature of insomnia is conditioned arousal affected by dysfunctional efforts to sleep and negative expectations, patients with insomnia become anxious and frustrated as insomnia symptoms persist. The mediator PSQI covers general sleep problems including sleep latency; sleep duration and efficiency; and sleep-disturbing factors, such as nocturia, nocturnal breathing problems, pain, feeling too hot or cold, use of sleeping pills, and daytime dysfunction. Even in the absence of direct insomnia-related factors, nearly all of these components are associated with somatic symptoms of depression (46), and more than half of patients with anxiety disorders report those somatic complaints (47). These observations are in agreement with the findings of our mediation analysis: depression and anxiety have indirect relationships with the degree of insomnia severity and poor sleep-related somatic symptoms, which in turn aggravates insomnia severity.

There was a systemic review on the relationships among anxiety, depression, and sleep disturbance. They showed that insomnia and sleep quality features have “bidirectional” relationships with anxiety and depression, respectively (48). Moreover, regarding treatment, cognitive behavioral therapy that focuses on attenuating anxiety and depression reduces insomnia severity and the symptoms of the two psychiatric conditions (49).

Our study is subject to several limitations. First, we did not consider comorbidities of anxiety and depression with other sleep disorders, including obstructive sleep apnea, narcolepsy, and restless legs syndrome. The presence of any pain or other significant medical illnesses was also not investigated. Second, even though we performed a population-based study with a low sampling error, the statistical power for the examination of subgroups might have been diminished due to the small samples (especially Group 3). Third, in the mediation analysis, we did not conduct a detailed investigation to elucidate the component of PSQI that features a close relationship with insomnia severity. Third, we did not analyze longitudinal data which causes a problem to establish causality, despite a quite large sample. Last, we didn't consider the impact of medication use when we analyzed our data. The medication status is an important factor that can change the subjects' mental status. Unfortunately, our research target was the general population. So, it was not easy to obtain optimal medical information from the survey method.

This report is, to the best of our knowledge, the first to evaluate how anxiety and depression affect sleep quality and the severity of insomnia. The prevalence of high risk for insomnia and the comorbidities with anxiety and depression is comparable to the findings of previous reports. Daytime sleepiness, general sleep quality, and insomnia severity were consistently poorer in subjects with both depression and anxiety. Also, we found that the effect of the combination of both psychiatric conditions was mediated by general sleep quality indices, which encompass insomnia and related somatic symptoms. We surmise that anxiety and depression affect insomnia in a supra-additive manner. When treating insomnia patients, clinicians should look for underlying comorbid psychiatric conditions to determine the appropriate therapy and enhance the therapeutic effect.

## Data Availability

The datasets analyzed in this manuscript are not publicly available. Requests to access the datasets should be directed to chumk@yuhs.ac.

## Author Contributions

C-MO, KC, and MC: study concept and design. C-MO, HK, KC, and MC: acquisition, analysis, and interpretation of data. C-MO and KC: drafting of the manuscript. HK, KC, and HN: statistical analysis. C-MO: obtained funding. KC and MC: study supervision. All authors read and approved the manuscript.

### Conflict of Interest Statement

The authors declare that the research was conducted in the absence of any commercial or financial relationships that could be construed as a potential conflict of interest.
